# Survival after Locoregional Treatments for Hepatocellular Carcinoma: A Cohort Study in Real-World Patients

**DOI:** 10.1100/2012/564706

**Published:** 2012-05-03

**Authors:** Simona Signoriello, Annalisa Annunziata, Nicola Lama, Giuseppe Signoriello, Paolo Chiodini, Ilario De Sio, Bruno Daniele, Giovanni G. Di Costanzo, Fulvio Calise, Graziano Olivieri, Vincenzo Castaldo, Rosario Lanzetta, Guido Piai, Giampiero Marone, Mario Visconti, Mario Fusco, Massimo Di Maio, Francesco Perrone, Ciro Gallo, Giovanni B. Gaeta

**Affiliations:** ^1^Department of Medicine and Public Health, Second University of Napoli, Via L. Armanni 5, 80138 Napoli, Italy; ^2^Department of Clinical and Experimental Medicine and Surgery “F. Magrassi-A. Lanzara”, Second University of Napoli, Via S. Pansini 5, 80131 Napoli, Italy; ^3^Rummo Hospital, Via dell'Angelo 1, 82100 Benevento, Italy; ^4^Cardarelli Hospital, Via A. Cardarelli 9, 80131 Napoli, Italy; ^5^National Cancer Institute, Via Mariano Semmola, 80131 Napoli, Italy; ^6^Moscati Hospital, Città Ospedaliera, Contrada Amoretta, 83100 Avellino, Italy; ^7^S. Anna and S. Sebastiano Hospital, Via F. Palasciano, 81100 Caserta, Italy; ^8^Ascalesi Hospital, Via Egiziaca a Forcella, 31 80139 Napoli, Italy; ^9^Incurabili Hospital, Via Maria Longo 50, 80138 Napoli, Italy; ^10^Campania Cancer Registry, Azienda Sanitaria Locale Napoli 3 sud, Piazza San Giovanni, 80031 Brusciano (NA), Italy

## Abstract

Evidence of relative effectiveness of local treatments for hepatocellular carcinoma (HCC) is scanty. We investigated, in a retrospective cohort study, whether surgical resection, radiofrequency ablation (RFA), percutaneous ethanol injection (PEI), and transarterial embolization with (TACE) or without (TAE) chemotherapy resulted in different survival in clinical practice. All patients first diagnosed with HCC and treated with any locoregional therapy from 1998 to 2002 in twelve Italian hospitals were eligible. Overall survival (OS) was the unique endpoint. Three main comparisons were planned: RFA versus PEI, surgical resection versus RFA/PEI (combined), TACE/TAE versus RFA/PEI (combined). Propensity score method was used to minimize bias related to non random treatment assignment. Overall 425 subjects were analyzed, with 385 (91%) deaths after a median followup of 7.7 years. OS did not significantly differ between RFA and PEI (HR 1.11, 95% CI 0.79–1.57), between surgery and RFA/PEI (HR 0.95, 95% CI 0.64–1.41) and between TACE/TAE and RFA/PEI (HR 0.88, 95% CI 0.66–1.17). 5-year OS probabilities were 0.14 for RFA, 0.18 for PEI, 0.27 for surgery, and 0.15 for TACE/TAE. No locoregional treatment for HCC was found to be more effective than the comparator. Adequately powered randomized clinical trials are still needed to definitely assess relative effectiveness of locoregional HCC treatment.

## 1. Introduction

Locoregional treatments are the mainstay of treatment of early stage hepatocellular carcinoma (HCC) [1–3]. Surgical resection should be particularly considered for patients with solitary tumours and well-preserved liver function. Transarterial embolization with (TACE) or without (TAE) chemotherapy is recommended for intermediate stage HCC patients who are ineligible for surgery or percutaneous ablation [1–3]. However evidence of relative effectiveness on survival of local treatments for hepatocellular carcinoma (HCC) is scanty due to the paucity of clinical trials and shortness of followup.

A meta-analysis [4] that compared surgery versus ablative treatments in the subgroup of tumors >3 cm found a survival benefit of surgery, but concluded that level of evidence was low and that further RCT were needed.

Two meta-analyses [5, 6], compared radiofrequency ablation (RFA) with PEI, and found a slight survival improvement with RFA over PEI, but relied upon few small trials with a short followup period and a limited number of events.

Three meta-analyses [7–9] assessed the efficacy of TACE/TAE versus supportive care, but ended up with contrasting results. Geschwind et al. failed to show a survival advantage versus supportive care alone and emphasised the poor quality of published trials [7]. Cammà et al. claimed that both TACE/TAE significantly reduced overall 2-year mortality, but the magnitude of benefit was relatively small [8]. Llovet and Bruix found that arterial embolization improved 2-year survival versus control, and this benefit was significant for TACE but not for TAE [9].

In general, these studies were conducted in specialized reference centers in well-selected patients. In this observational cohort study we assessed the relative effectiveness on long-term survival of locoregional treatments for HCC in real-world patients.

## 2. Patients and Methods

### 2.1. Study Subjects

 The study had a retrospective cohort design. All patients first diagnosed with HCC (ICD-9 155.0) and treated with any locoregional therapy from 1998 to 2002 in public hospitals of Campania, southern Italy, were potentially eligible. Potential patients were retrieved from the Discharge Information System of the Regional Health Service; eligibility criteria were subsequently checked by perusing clinical records. Child-Pugh score C, presence of portal vein thrombosis, massive tumour morphology and liver involvement greater than 50% were exclusion criteria. Time interval was chosen “a priori” to allow an adequate followup.

The study protocol was approved by the ethic committees of all the participating Institutions.

### 2.2. Endpoint and Covariates

Overall survival (OS) was the unique outcome measure and was defined as the time from the date of the first local intervention until death for any cause or until date of last followup. Date of death was ascertained by the administrative registry offices of patients' towns of residence.

Baseline demographic, clinical, and tumour-related variables were derived from clinical records. The CLIP prognostic score [10, 11], used for statistical adjustment, was calculated “a posteriori” from information reported in clinical records. Performance status was very rarely reported so that the Barcelona Clinic Liver Cancer [3] staging could not be assessed.

### 2.3. Statistical Analysis

Three main comparisons were planned, RFA versus PEI, surgery versus RFA or PEI, TACE/TAE versus RFA or PEI.

To minimize biases related to nonrandom assignment we used the propensity score method [12–14], where the relationship between treatment and survival is adjusted for patient's likelihood of receiving that therapy given his/her prognostic profile.

For each comparison the primary multivariable analysis was performed by a Cox proportional hazard model with compared treatments and propensity score as covariates, stratified by the number of missing values in the CLIP score components. Propensity score was estimated for each comparison by a logistic regression model that included, as covariates, age, sex, CLIP prognostic score, and number of missing components of the CLIP score [15]. Only subjects with overlapping values of propensity score were analysed for each main comparison. Proportional hazard assumption was checked by graphical inspection [16].

As a sensitivity analysis, further statistical models were performed to assess the consistency of results [17–20]: (i) modelling propensity score with cubic regression splines in order to obviate the need for assuming a linear effect [17], (ii) stratifying the model by subclasses defined by propensity score quintiles [18], (iii) weighting each subject by the inverse of the individual probability of receiving the treatment assumed, estimating variance via the empirical sandwich method [19], and (iv) substituting CLIP score in the Cox model with its components [20].

 Unadjusted cumulative survival curves were depicted by Kaplan-Meier (K-M) method and compared by the Mantel-Haenszel test (MH) and Peto and Peto modification of the Wilcoxon rank sum test (WPP). The two tests give different weights to events, the second one giving more weight to earlier events.

Since guidelines [1, 2] suggest that treatments could have different effects in particular subgroups of subjects, for each comparison we repeated analyses in predefined subgroups of subjects.

All analyses were performed with R software, version 2.9.1 (Development Core Team. R: A Language and Environment for Statistical Computing. R Foundation for Statistical Computing. 2009).

## 3. Results

**Figure 1:**
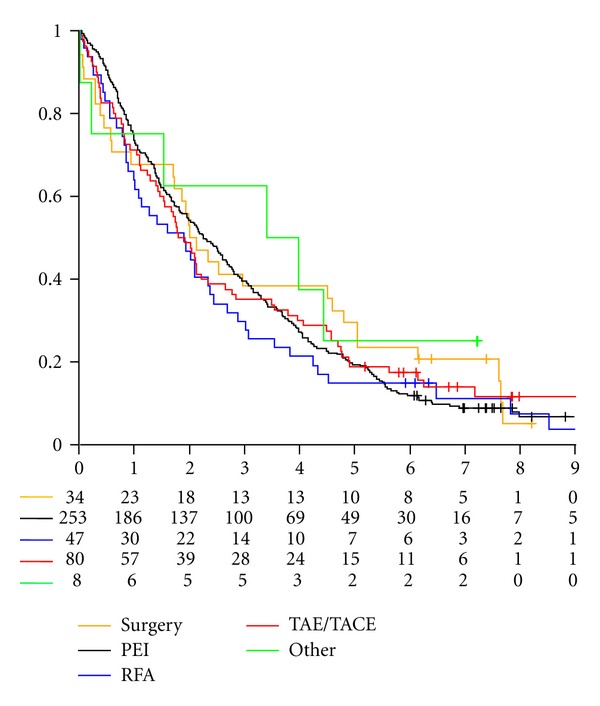
Kaplan-Meier cumulative survival curves for the study treatments.

Overall 441 HCC patients discharged from January 1998 and December 2002 were eligible. Sixteen cases were excluded because of lack of any follow-up information, thus the final study sample involved 425 subjects. Baseline characteristics of the 425 patients are reported in Table 1. PEI was the most common treatment (60%) followed by TACE/TAE (19%), while surgical resection and RFA were performed in fewer subjects. Three patients received at the same time both PEI and RFA and were excluded only from the comparison of PEI versus RFA; eight patients received other local treatments (laser therapy) and were excluded from all comparisons. On the whole, prognostic factors did not differ a lot among treatments, although seemingly surgery was performed in patients with a more favourable prognostic profile, and TACE/TAE in patients with more severe disease. Overall, after a median follow-up time of 7.7 years, 385 (91%) deaths were registered. Observed cumulative survival curves for all study treatments are depicted in Figure 1.

In Table 2 we reported results of the three multivariable primary analyses. No significant difference in overall survival was found for any of the three planned comparisons.

Hazard ratio (HR) of RFA versus PEI was equal to 1.11 (95% C.I. 0.79 to 1.57, *P* = 0.53). Estimated probabilities to be alive at 5 years were equal to 0.14 (95% CI 0.07 to 0.27) and 0.18 (95% CI 0.14 to 0.23) for RFA and PEI, respectively. At the univariate analysis, both MH test (*P* = 0.36) and WPP test (*P* = 0.14) were not statistically significant (Figure 1).

HR of surgery versus the two percutaneous ablation therapies combined was equal to 0.95 (95% C.I. 0.64 to 1.41, *P* = 0.79). Estimated probabilities to be alive at 5 years were equal to 0.27 (95% CI 0.16 to 0.46) and 0.17 (95% CI 0.13 to 0.22) for surgery and RFA/PEI, respectively. At the univariate analysis both MH (*P* = 0.52) and WPP test (*P* = 0.94) were not statistically significant (Figure 1).

HR of TACE/TAE versus the two percutaneous ablation therapies combined was equal to 0.88 (95% C.I. 0.66 to 1.17, *P* = 0.38). Estimated probabilities to be alive at 5 years were equal to 0.15 (95% CI 0.10 to 0.24) and 0.17 (95% CI 0.13 to 0.22) for TACE/TAE and RFA/PEI, respectively. At the univariate analysis MH test did not reveal differences between arms (*P* = 0.44) while WPP test (*P* = 0.03) was statistically significant (Figure 1), thus highlighting an earlier prognostic advantage for RFA/PEI that later disappeared.

Superimposable results were found for all comparisons at sensitivity analyses, where other adjustment modalities were applied (Table 3).

**Figure 2:**
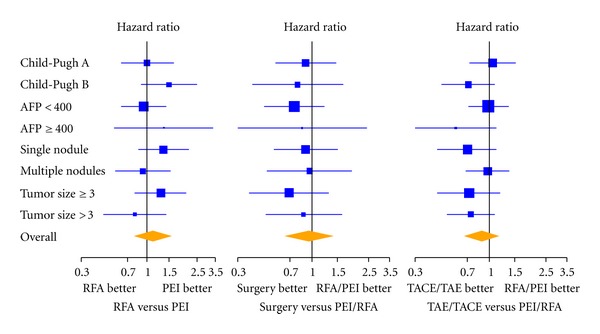
Univariate comparisons of RFA versus PEI (left panel), surgical resection versus RFA/PEI (middle panel) and TACE/TAE versus RFA/PEI (right panel) within major patient subgroups. The area of each square is proportional to the size of the subgroup; horizontal lines depict 95% confidence intervals of the hazard ratio estimates.

Results of univariate analyses in predefined subgroups of subjects for the three comparisons are reported in Figure 2. For every comparison, results in the study subgroups were similar to the overall analyses without any evidence of heterogeneity.

## 4. Discussion

This observational study in a clinical practice setting did not find survival differences between local treatments in any of the study comparisons. Clearly, robust evidence of treatment efficacy may only result from adequately powered randomized trials and observational studies may be flawed by several shortcomings. However, our findings add a notable piece of information to the literature of locoregional treatments for HCC, where only small clinical trials, if any, are available and are usually performed in specialized reference centers on well-selected patients.

In this study we addressed the potential biases of observational studies in several ways. First, we pursued a population-based approach, identifying the reservoir of potentially eligible patients from an independent source (the Discharge Information System of the Campania Regional Health Service), thus reducing the risk of selection bias. In addition we chose survival as the unique endpoint of the study, as recommended when effectiveness between therapies is assessed [1, 2]. To remove the ascertainment bias, the date of death was independently derived from the administrative death registries. We were unable to assess the outcome only in 16 subjects because of migration or mistakes in residence information. Finally, we counteracted indication bias (i.e., patients' selection for different therapies) by adjusting comparisons for propensity score [12–14], (i.e., the probability of receiving a given therapy conditionally on the patient's individual prognostic profile).

A major strength of our study is the length of followup with a large number of deaths observed (91% of the whole sample), that allowed a complete picture of the survival experience of the study cohort. To our knowledge, our cohort is the largest reported in the literature for this kind of study, after the one of Arii et al. who used a population-based approach starting from a nationwide survey in Japan [21]. Furthermore, from a methodological viewpoint, we assessed whether results persisted under possible violations of the statistical assumptions, by repeating the analyses with several adjustment modalities. The consistency of results across different models reinforces their validity, although some residual confounding could still be present, due to unknown covariates not included in the models [22, 23].

We adjusted for missing information in multivariable analyses, but we acknowledge that missing data might partially affect our findings. Furthermore we only assessed first-line local treatments, since information on successive treatments was largely unreliable.

The major and unexpected finding of our results was the lack of significant differences even in univariate analyses, where we expected survival differences at least as a consequence of indication bias. Actually patients' baseline characteristics overlapped substantially among treatments, despite the careful selection recommended by the international guidelines [1, 2]. Although this might be partly explained by the fact that our cohort was antecedent to guidelines, an alternative explanation is that the choice of local treatment was rather driven by clinicians' preferences or availability of skills.

Although our results may appear surprising, they mirror some uncertainties of the literature results. Two meta-analyses [5, 6] analyzed the comparisons of RFA versus PEI and found a significant survival improvement favouring the former over the latter one, while a systematic review on the same trials concluded that data does not provide enough evidence to support survival benefits coming from RFA [24]. The five randomized trials that tested the two percutaneous treatments and were considered in the meta-analyses [5, 6] were small and had a short follow-up. Interestingly, we did not find any difference between RFA and PEI even in the subgroups of patients (like those with larger tumor size) in which international guidelines claim that RFA should be more effective than PEI [2].

 Surgery has been compared to percutaneous ablation in three small randomized trials [25–27]. Huang et al. [25] and Chen et al. [26] did not find significant benefits of surgery, while Huang et al. [27] found that surgical resection increased overall survival in patients who met the Milan criteria. A meta-analysis [4] that included only one randomized trial and several observational studies, found a survival benefit of surgery versus ablative treatments in the subgroup of tumors >3 cm, but concluded that level of evidence was low and that further RCT were needed to define the relative value of surgery and RFA. Unfortunately in our study the number of surgical resections, that were performed only in two big Institutions, is small and comparison is underpowered. However we did not find any difference even in the subgroups of patients with single nodules or Child-Pugh A, that is, the best candidates to resection [2].

To our knowledge TACE/TAE alone have never been compared with other locoregional treatments since guidelines consider TACE/TAE as restricted to ‘nonsurgical HCC that are also ineligible for percutaneous ablation [2]. As expected, we found slightly worse patients in the TACE/TAE group, but in multivariable analysis we were unable to find any difference in long-term survival from RFA/PEI, neither overall nor in selected subgroups.

In conclusion, although our approach does not allow definitive statements, our results show that, in a real-world setting, uncertainties in the choice and in the outcome of local treatments of HCC are still present. Educational projects and population-based observational studies, supported by well-planned RCTs, are still needed to define the relative effectiveness of locoregional treatments.

## Figures and Tables

**Table 1:** Baseline characteristics of the study patients by treatment. Data are reported as absolute numbers (percentages), but for age and AFP.

Variable	Total (*N* = 425)	Surgery (*N* = 34)	PEI° (*N* = 256)	RFA° (*N* = 50)	TAE/TACE (*N* = 80)	Other^∧^ (*N* = 8)
*Year of diagnosis*						
1998	33 (8%)	4 (12%)	23 (9%)	2 (4%)	4 (5%)	0 (0%)
1999	83 (20%)	5 (15%)	55 (21%)	9 (18%)	14 (18%)	0 (0%)
2000	106 (25%)	12 (35%)	59 (23%)	9 (18%)	26 (32%)	1 (13%)
2001	112 (26%)	7 (21%)	68 (27%)	16 (32%)	21 (26%)	1 (13%)
2002	91 (21%)	6 (18%)	51 (20%)	14 (28%)	15 (19%)	6 (75%)

Male gender	327 (77%)	30 (88%)	188 (73%)	40 (80%)	65 (81%)	7 (88%)

Age *yrs*, mean (SD)	67 (8)	62 (7)	68 (7)	67 (8)	64 (8)	68 (7)

*Diagnostic assessment*						
Histology	195 (46%)	23 (68%)	125 (49%)	13 (26%)	32 (40%)	3 (38%)
Instrumental + AFP > 200	51 (12%)	3 (9%)	28 (11%)	9 (18%)	12 (15%)	0 (0%)
Instrumental only	179 (42%)	8 (24%)	103 (40%)	28 (56%)	36 (45%)	5 (63%)

*Instrumental appraisal*						
Ultrasonography	187 (44%)	11 (32%)	123 (48%)	21 (42%)	27 (34%)	6 (75%)
NMR	13 (3%)	4 (12%)	4 (2%)	5 (10%)	0 (0%)	0 (0%)
CT	178 (42%)	14 (41%)	96 (38%)	20 (40%)	48 (60%)	2 (25%)
Missing	47 (11%)	5 (15%)	33 (13%)	4 (8%)	5 (6%)	0 (0%)

*Underlying liver disease*						
Liver cirrhosis	414 (97%)	32 (94%)	250 (98%)	49 (98%)	78 (98%)	8 (100%)
Chronic Hepatitis	11 (3%)	2 (6%)	6 (2%)	1 (2%)	2 (2%)	0 (0%)

*Etiology*						
Viral	366 (86%)	32 (94%)	214 (83%)	46 (92%)	71 (89%)	6 (75%)
Nonviral	10 (2%)	0 (0%)	8 (3%)	0 (0%)	2 (2%)	0 (0%)
Missing	49 (12%)	2 (6%)	34 (13%)	4 (8%)	7 (9%)	2 (25%)

*Viral etiology*						
HCV	324 (76%)	24 (71%)	197 (77%)	43 (86%)	56 (70%)	7 (88%)
HBV	59 (14%)	9 (26%)	21 (8%)	11 (22%)	18 (22%)	1 (13%)

*Child-Pugh score*						
A	200 (47%)	22 (65%)	105 (41%)	26 (52%)	42 (52%)	7 (88%)
B	137 (32%)	8 (24%)	79 (31%)	20 (40%)	31 (39%)	0 (0%)
Missing	88 (21%)	4 (12%)	72 (28%)	4 (8%)	7 (9%)	1 (12%)

AFP *mg/dl,* median (IQ range)	27 (7–156)	12 (3–146)	28 (8–127)	34 (9–183)	31 (7–334)	14 (5–27)

*Number of nodules*						
<4	305 (72%)	25 (74%)	189 (74%)	38 (76%)	49 (61%)	5 (62%)
≥4	12 (3%)	0 (0%)	3 (1%)	1 (2%)	8 (10%)	0 (0%)
Missing	108 (25%)	9 (26%)	64 (25%)	11 (22%)	23 (29%)	3 (38%)

*Tumour size (cm)*						
≤3	207 (49%)	13 (38%)	143 (56%)	24 (48%)	23 (29%)	5 (62%)
3–5	90 (21%)	9 (26%)	43 (17%)	11 (22%)	28 (35%)	0 (0%)
>5	39 (9%)	4 (12%)	12 (5%)	7 (14%)	15 (19%)	1 (12%)
Missing	89 (21%)	8 (24%)	58 (23%)	8 (16%)	14 (18%)	2 (25%)

*Tumour morphology*						
Single nodule	204 (48%)	18 (53%)	132 (52%)	23 (46%)	26 (32%)	5 (62%)
Multiple nodules	175 (41%)	10 (29%)	93 (36%)	24 (48%)	48 (60%)	3 (38%)
Missing	46 (11%)	6 (18%)	31 (12%)	3 (6%)	6 (8%)	0 (0%)

*CLIP score *						
0	76 (18%)	9 (26%)	43 (17%)	10 (20%)	12 (15%)	2 (25%)
1	111 (26%)	10 (29%)	65 (25%)	13 (26%)	23 (29%)	1 (12%)
2	59 (14%)	3 (9%)	29 (11%)	9 (18%)	19 (24%)	1 (12%)
3	14 (3%)	0 (0%)	6 (2%)	3 (6%)	5 (6%)	0 (0%)
>3	0 (0%)	0 (0%)	0 (0%)	0 (0%)	0 (0%)	0 (0%)
Missing	165 (39%)	12 (35%)	113 (44%)	15 (30%)	21 (26%)	4 (50%)

°Including three subjects who received both RFA and PEI; ^∧^Including 8 laser therapy; PEI: percutaneous ethanol injection, RFA: radiofrequency ablation, TACE/TAE transarterial embolization with (TACE) or without (TAE) chemotherapy, AFP: alphafetoprotein, NMR nuclear magnetic resonance.

**Table 2:** Effectiveness of locoregional treatment on overall survival in Cox proportional hazard model adjusted by propensity score.

Model	HR (95% CI)	*P*
RFA versus PEI (*n* = 47 + 239)	1.11 (0.79–1.57)	0.53
Surgery versus RFA/PEI (*n* = 34 + 255)	0.95 (0.64–1.41)	0.79
TAE/TACE versus RFA/PEI (*n* = 80 + 287)	0.88 (0.66–1.17)	0.38

PEI: percutaneous ethanol injection, RFA radiofrequency ablation, TACE/TAE: transarterial embolization with (TACE) or without (TAE) chemotherapy.

**Table 3:** Effectiveness of locoregional treatment on overall survival in Cox proportional hazard model adjusted by propensity score. Sensitivity analysis.

Model	HR (95% CI)	*P*
*RFA versus PEI (n* = 47 + 253)		
Adjustment by propensity score (*n* = 47 + 239)		
Linear	1.11 (0.79–1.57)	0.53
Cubic spline	1.09 (0.77–1.54)	0.63
Stratified (quintiles)	1.11 (0.78–1.58)	0.56
Inverse probability weighting	1.13 (0.82–1.57)	0.46
Adjustment by prognostic covariates (*n* = 47 + 253)	1.21 (0.87–1.71)	0.25

*Surgery versus RFA/PEI *(*n* = 34 + 303)		
Adjustment by propensity score (*n* = 34 + 255)		
Linear	0.95 (0.64–1.41)	0.79
Cubic spline	0.95 (0.64–1.41)	0.81
Stratified (quintiles)	0.95 (0.61–1.48)	0.82
Inverse probability weighting	0.80 (0.52–1.24)	0.32
Adjustment by prognostic covariates (*n* = 34 + 303)	0.96 (0.64–1.42)	0.82

*TAE/TACE versus RFA/PEI *(*n* = 80 + 303)		
Adjustment by propensity score (*n* = 80 + 287)		
Linear	0.88 (0.66–1.17)	0.38
Cubic spline	0.89 (0.67–1.18)	0.41
Stratified (quintiles)	0.86 (0.65–1.15)	0.32
Inverse probability weighting	0.98 (0.73–1.32)	0.90
Adjustment by prognostic covariates (*n* = 80 + 303)	0.83 (0.63–1.10)	0.20

PEI: percutaneous ethanol injection, RFA: radiofrequency ablation, TACE/TAE: transarterial embolization with (TACE) or without (TAE) chemotherapy.

## Appendix

### Investigators from the Participating Institutions

Cardarelli Hospital, Napoli (G. Di Costanzo, F. Calise, F. Paradiso, L. Staiano); National Cancer Institute, Napoli (F. Perrone, M. Di Maio, G. Olivieri, V. Molese), Incurabili Hospital, Napoli (M. Visconti, A. Salvio, L. De Paola); Pellegrini Hospital, Napoli (A. Guaragna); Ascalesi Hospital, Napoli (G. Marone); Loreto nuovo Hospital, Napoli (W. Longanella); Second University of Napoli: Infectious diseases (GB Gaeta, M. Stanzione, G. Stornaiuolo), Internal medicine (L. Rinaldi); Gastroenterology (I. De Sio); Surgery (G. Galizia); S. Sebastiano Hospital, Caserta (G. Piai, A. Annunziata, T. Sgueglia); Moscati Hospital, Avellino (V. Castaldo); Rummo Hospital, Benevento (B. Daniele, R. Lanzetta); District Hospital, Aversa (B. Russo).
